# Neurons, Glia, Extracellular Matrix and Neurovascular Unit: A Systems Biology Approach to the Complexity of Synaptic Plasticity in Health and Disease

**DOI:** 10.3390/ijms21041539

**Published:** 2020-02-24

**Authors:** Ciro De Luca, Anna Maria Colangelo, Assunta Virtuoso, Lilia Alberghina, Michele Papa

**Affiliations:** 1 Laboratory of Morphology of Neuronal Network, Department of Public Medicine, University of Campania “Luigi Vanvitelli”, 80138 Napoli, Italy; delucaciro88@gmail.com (C.D.L.); assunta-1989@hotmail.it (A.V.); michele.papa@unicampania.it (M.P.); 2 Laboratory of Neuroscience “R. Levi-Montalcini”, Dept. of Biotechnology and Biosciences, University of Milano-Bicocca, 20126 Milano, Italy; 3 SYSBIO Centre of Systems Biology ISBE.ITALY, University of Milano-Bicocca, 20126 Milano, Italy; lilia.alberghina@gmail.com

**Keywords:** glia, tripartite synapse, synaptic plasticity, neurovascular unit, systems biology

## Abstract

The synaptic cleft has been vastly investigated in the last decades, leading to a novel and fascinating model of the functional and structural modifications linked to synaptic transmission and brain processing. The classic neurocentric model encompassing the neuronal pre- and post-synaptic terminals partly explains the fine-tuned plastic modifications under both pathological and physiological circumstances. Recent experimental evidence has incontrovertibly added oligodendrocytes, astrocytes, and microglia as pivotal elements for synapse formation and remodeling (tripartite synapse) in both the developing and adult brain. Moreover, synaptic plasticity and its pathological counterpart (maladaptive plasticity) have shown a deep connection with other molecular elements of the extracellular matrix (ECM), once considered as a mere extracellular structural scaffold altogether with the cellular glue (i.e., glia). The ECM adds another level of complexity to the modern model of the synapse, particularly, for the long-term plasticity and circuit maintenance. This model, called tetrapartite synapse, can be further implemented by including the neurovascular unit (NVU) and the immune system. Although they were considered so far as tightly separated from the central nervous system (CNS) plasticity, at least in physiological conditions, recent evidence endorsed these elements as structural and paramount actors in synaptic plasticity. This scenario is, as far as speculations and evidence have shown, a consistent model for both adaptive and maladaptive plasticity. However, a comprehensive understanding of brain processes and circuitry complexity is still lacking. Here we propose that a better interpretation of the CNS complexity can be granted by a systems biology approach through the construction of predictive molecular models that enable to enlighten the regulatory logic of the complex molecular networks underlying brain function in health and disease, thus opening the way to more effective treatments.

## 1. Introduction

Neuronal synapses are, at a biochemical level, stations of electrochemical signaling between the dynamic circuits underlying the complex and deeply interconnected processes of motor and learning functions. Intricate as brain processing may seem, the synapse and its plasticity represent the anatomic and functional unit that can explain it. Hundreds of proteins form the synaptic elements, and their correct expression, structural organization, turnover, and reshaping capability are pivotal for the proper function of the central nervous system (CNS) [1,2].

The pivotal characteristic of the brain is the continuous and strategic ability to modify itself in an experience-based fashion. The subsequent behavior could rely on the strength of circuit transmission and the reinforcing of active synapses or the pruning of new ones. Although development and adulthood show different patterns of synaptic plasticity, this function is fundamental for brain homeostasis [3,4]. Single-cell or matrix contribution could not be easily dissected; however, in the last decades, numerous studies have emerged to enroll glia in the first paradigm shift model, the tripartite synapse including the astrocytes [5]. Indeed, neuronal activity can be controlled by astrocytes in their different and specialized morphologies (i.e., protoplasmic, fibrous, perivascular, and Bergman glia) [6,7]. Higher functions and distinctive neurological competences of the human brain have also been associated with differences between humans and other mammals regarding glial cells and their pattern of gene expression, cellular morphology, and peculiar calcium dynamics [8]. Moreover, it is now clear that oligodendrocytes and microglia also contribute to synaptic plasticity. Oligodendrocytes have shown the potential role of signaling transducers and builder of the extracellular environment [9]. Microglia, instead, in addition to their role of specialized resident macrophage of the CNS, has shown to interact with neurons, to assist their formation in the neural niche and to guide circuit integration and tuning (axonal growth, dendritic sprouting, synapse remodeling) [10].

Finally, the extracellular matrix (ECM), acting as a functional scaffold, represents almost one-fifth of the brain volume. The complex network constituted by proteoglycans, glycoproteins, and glycosaminoglycan sustains neuronal function and provides, together with structural support, a reservoir of trophic factors, signaling molecules, biochemical pathways and long-distance gradient-like communication between cellular components of the CNS [11,12].

The fourth compartment of the synapse is indeed a non-cellular element [13]. The resilience of CNS and synaptic plasticity in the critical period of development and in the adult brain depends on specialized forms of ECM, such as the interstitial matrix, the perineural nets (PNNs) and the basement membrane. Particularly, this last structure is important for the integration of the neurovascular unit (NVU) to obtain an overall model that could be used as a start point for a systems biology-based approach [14]. The implementation of protein–protein interactions involving all the cellular and non-cellular elements of the system can help the building of hub-spoke network maps [14] to design further experiments with translational purposes. Indeed, the ECM is involved in the bidirectional exchange of nutrients and metabolic products between CNS and systemic circulation. The specialized blood–brain barrier (BBB) is one of the finest *exempla* of integration among cellular compartments (glia, pericytes, endothelium) and the ECM, that can selectively permit the transmembrane active transport, the diffusion of molecules through tight junctions, and the selective loosening and remodeling of the BBB [15]. The matrix metalloproteinases (MMPs), as well as other proteases and their relative matrix receptors and regulators, can actively participate in the modulation of CNS circuitry response to various stimuli. In addition, they can mediate the immune system activation and the reshaping of the NVU [16]. This complex and emergent system is furthermore pivotal in the so-called glymphatic regulation, a novel physiological model to clear out wastes of the cellular metabolism from the CNS parenchyma through the dynamic exchange between cerebrospinal fluid (CSF) and the ECM via the NVU [17,18].

In consideration of the great complexity of the synapse organization (defined as penta-partite if we take into account ECM and NVU), here we aim to construct a model of the synapse that can be used for a systems biology modeling. This approach can help to gain new insights into pathogenetic mechanisms underlying complex molecular processes, such as cancer and neurodegenerative disorders. For instance, this strategy is being used to integrate computational models and metabolic flux analysis in cancer cells and make prediction of metabolic reprogramming underlying cancer cell growth [19]. Computational studies of networks of genes and pathways in Alzheimer’s and Parkinson’s disease (PD) were also effective in identifying functional and topological similarities and differences between the two pathologies [20]. In addition, a modeling strategy has been used to construct a map of pathogenetic processes and pathways involved in PD [21]. Submodules of this map are currently used to unravel specific pathways and their interconnection with interacting processes. For instance, based on experimental evidence, we are currently implementing a mathematical model that exploits the ROS management system and its connection with the metabolism, as well as the relevance of ROS-mitochondria remodeling in neuronal differentiation and maintenance of the neuronal phenotype, neuroprotection, and antigliosis [22,23]. A novel computational model could be used to develop differential neuroprosthetic stimulation modulating pain processing [24]. Once validated, these mathematical models can be useful to predict the impact of any perturbation (genetic or environmental) on the complex biological process(es) under investigation. This could have many positive outcomes in terms of drug discovery and personalized medicine, as it can favor the identification of effective targets for functional recovery.

**Figure 1:**
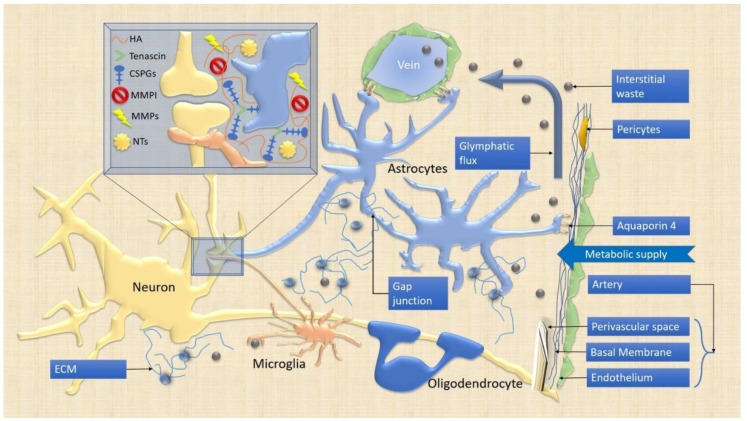
Schematic representation of the synaptic cleft. The main cellular and extracellular components implicated in both physiological and pathological synaptic changes are schematically represented. Oligodendrocytes ensure the correct myelination of the circuits. Microglia constantly scan the microenvironment and remove the debris. From the perivascular space of penetrating arteries, and the neurovascular unit (NVU) elements, fluid dynamics convey waste products toward perivenous spaces and control metabolic supply. The influx-efflux is regulated by Aquaporin-4 (AQP4) water channels densely expressed within astrocyte end-feet. The synaptic cleft magnified in the blue box in the high-left corner encompasses both glial and neuronal elements tightly connected through the extracellular matrix (ECM). The ECM functional scaffold composed of tenascin, hyaluronic acid (HA) and chondroitin sulfate proteoglycans (CSPGs) regulates the expression of neurotrophins (NTs), matrix metalloproteinases (MMPs), and their inhibitors (MMPI).

Impairment of the complex multicellular and multimolecular synaptic system induces acute or chronic CNS pathologies due to the dysfunction of any of these synaptic components with the consequent domino effect. To better understand how to favor the maintenance of adaptive plasticity, it would be useful to construct molecular models able to enlighten the regulating logic of the complex molecular network, which belongs to different cellular and subcellular domains. To this end, we will discuss in detail (***i***) the interactions between cellular elements in the synaptic cleft, (***ii***) how glial cells can modulate synaptic plasticity, and (***iii***) the role of interstitial ECM and the NVU in both physiological (adaptive) and pathological (maladaptive) circumstances (Figure 1). For each cellular and molecular component, we will consider some of the main molecular pathways that should be taken into account when considering the entire system as an interconnected unit.

## 2. The Synaptic Cleft

Synaptic transmission is a highly specialized process. The punctual description of different types of neuronal cells with their various morpho-functional phenotypes goes beyond the aim of this review, thus only common features will be highlighted to describe the proposed system biology approach. Specific proteins organized in the synaptic cleft allow communication between neurons, rapidly and effectively through transmitter secretion [25]. The storage of transmitters inside the vesicles is a highly selective and energy-consuming task, with the employment of transporters, ion channels and the ATPase protonic pump that uses ATP to supply the proton gradient essential for vesicles loading with neurotransmitter [26]. The action potential is conducted through the axon voltage-gated channels leading to the increase of calcium concentration and the phosphorylation of synapsin that releases the tethering of vesicles to the cytoskeleton and permits the formation of the molecular machinery responsible for vesicle fusion with the cell membrane, the (soluble N-ethylmaleimide sensitive factor attachment protein -SNAP- receptor) SNARE complex [27].

The SNARE complex consists of (Vesicle-Associated Membrane Proteins) VAMPs, linked to the vesicular (v) membrane, the so-called v-SNAREs, and the cellular target (t)-SNAREs composed of the synaptosomal nerve-associated protein 25 (SNAP-25) and syntaxin-1, with a palmitoyl anchor to the pre-synaptic inside membrane [27]. VAMPs consist of seven recognized family members including VAMP1/2 (also known as synaptobrevin 1/2), VAMP4 and VAMP3/5/7/8 (also known as cellubrevin, myobrevin, Tetanus insensitive VAMP and endobrevin) [28]. To exert their role, these proteins are largely present on the cytoplasmic side of the vesicles and cellular membranes [29]. VAMP1/2 are the most abundant in the CNS, particularly expressed in neuronal vesicles, although also recognized on glandular secretory cells [27].

The fusion pore for vesicle secretion is the most controversial of the described mechanisms due to its heterogeneity and dynamicity. Indeed, various genes associated with the synapse have been implicated in neurological and psychiatric diseases, and their expression varies across brain areas, being modified by different cellular elements [30,31,32]. The difficulty of studying the complex protein–protein interactions is due to the inability of in vitro or in vivo conventional imaging to visualize multiple protein species in one intact sample with a high resolution of their sub-synaptic organization [33]. The probe-based imaging for sequential multiplexing (PRISM) methodology seems to be very versatile to obtain high resolution and dynamic visualization of multiple proteins interactions with reduced background fluorescence and the simultaneous immunostaining of an intact sample [34]. This technique could be useful to better investigate the synapse since it can screen protein interactions leading to maladaptive phenotypes and can count on multiple-level protein networks (e.g., 12 synaptic targets and 66 pair-wise synaptic co-localizations) in normal or perturbed cultures with high spatial or temporal resolution [34].

Synaptophysin (Syp) has been proposed as the initiator of the fusion pore, its role remaining however not utterly accepted [26,35]. The v-SNARE/t-SNARE interaction seems mandatory to generate the anchoring site for the pore formation [27]. These proteins are the target of the light chain of botulinum neurotoxins (BoNTs), the most toxic bacterial toxins produced by the anaerobic, spore-forming *Clostridium* (*C.*) species (i.e., *C. botulinum*, *C. butyricum*, and *C. baratii*) [36]. The importance of SNARE proteins is practically the reason for the astonishing toxicity of BoNTs (median lethal dose (LD50): 1 ng/kg, intraperitoneally) [37]. The first considerations about the role of BoNTs in medicine were focused on the peripheral release of acetylcholine at the neuromuscular junction (NMJ) [38]. However, it was reported that subcutaneous administration of BoNTs could reduce synaptic transmission and vesicle release of neurotransmitters facilitating vasodilation and pain-sensitization (e.g., Calcitonin Gene-Related Peptide), thus interfering with not only the NMJ but also the trigeminovascular system [39] or with oxidative-stress production [40].

**Figure 2:**
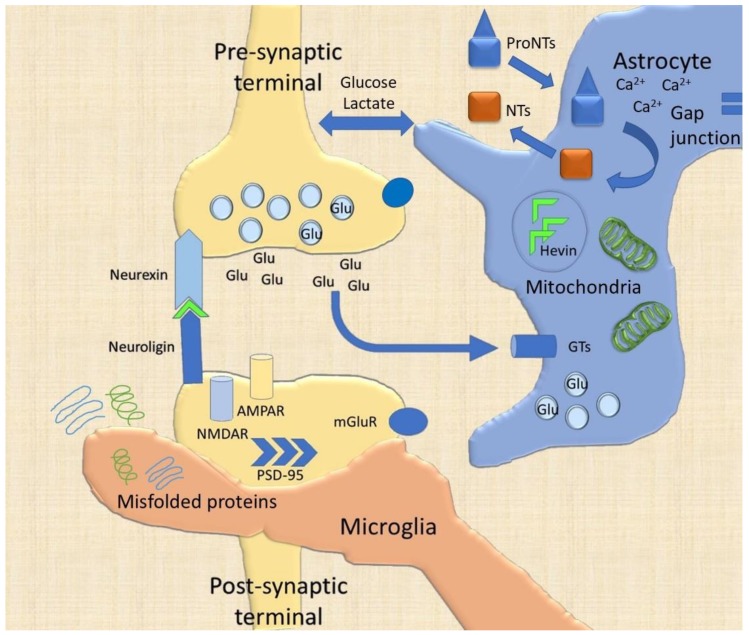
Cellular elements in the synaptic cleft. The schematic representation of a glutamatergic synapse highlights the role of molecular pre-synaptic (neurexins), post-synaptic (neuroligin) proteins and the astrocytic hevin in the stabilization of the cleft structure. The reuptake of neurotransmitters by glutamate transporters (GTs) is mainly provided by astrocytes. Moreover, astrocytes are responsible for the proneurotrophins (proNTs) alternative intracellular processing to active NTs and the metabolic coupling. The synaptic plasticity phenomena are widened by the astrocytic calcium waves, ensured through gap junction and by microglial trogocytosis (the partial engulfment of dendritic spines or axonal portions). The scavenger role of microglia is nonetheless necessary to avoid waste accumulation and synaptic failure.

The release of vesicles is a fixed, all-or-none process. The increase of intracellular calcium concentration through voltage-gated ionotropic channels is essential to trigger quantal exocytosis [27]. The calcium-binding protein synaptotagmin is necessary to facilitate the binding of phospholipids on the cytoplasmic side of the membrane [41]. The described process allows the neurotransmitters to be released in the synaptic cleft; however, the complexity of CNS response could not rely on a simple all-or-none quantal exocytosis that is indeed finely-tuned by the glial cells (Figure 2) [41]. The most common synapse in the cerebral cortex (neocortex and hippocampal allocortex) is between the axon of a presynaptic cell and the dendrite of a postsynaptic cell (axodendritic synapses). The axodendritic synapses of some neurons are located on the spines, highly specialized structures, protruding from the dendritic trunk, localizing the specific connection and increasing the density of synaptic terminals [42]. Electron microscopy further recognized the presence in certain synapses (known as asymmetric or type I synapses) of electron-dense structures called postsynaptic densities (PSD). These PSD where recognized prevalently in excitatory synapses and encompass anchorage proteins to the cytoskeleton, postsynaptic receptors and associated signaling transducers (Figure 2) [43]. The hundreds of proteins inside the PSD are interconnected and function both as a scaffold and as transmitting complexes with the ability to interact with each other and form heteromeric structures.

The most represented protein of the PSD is known as synapse-associated protein 90 (SAP-90) or PSD-95 (based on its molecular weight). PSD-95 is part of the scaffold family proteins called membrane-associated guanylate guanyl kinases (MAGUK) and has been associated with the increase of dendritic spines, regulation of neurotransmitter receptors and synapse stabilization and plasticity [43]. Other scaffold proteins include Homer and SHANK (SH3 and multiple ankyrin-repeat domains) families [43].

On the other hand, symmetric or type II synapses have slight electron-dense postsynaptic structures and are mainly inhibitory [44]. This classical division is schematic and oversimplified, but useful to comprehend the functioning of the synaptic cleft in general. As stated before, a clear and utter understanding of protein–protein interaction at synaptic level needs to be further elucidated with novel methods that eventually will reveal a fine-tuned complexity, with multiple specialized forms of both immature or mature synapses depending on brain areas, functional state, involved neurotransmitters, and even pathological responses.

The presynaptic neurexins and their relative neuroligin ligands on the postsynaptic terminal are nonetheless important for the formation, maturation and stabilization of synapses and for the interaction with glial cells and the ECM (Figure 1 and Figure 2). Neuroligins 1–4 seem to be unessential for synapse assembly in vivo, but they are pivotal for its maturation and proper functioning [41]. Their interactions with neurexins (α and β) affect both type I and type II synapses and the recruitment of other scaffolding proteins and receptors [45,46]. Mutations of these genes (NRXN1/2/3, NLGN1/3/4), together with SHANK family proteins, have been associated with autistic spectrum disorders (ASD) and schizophrenia [47].

Neurotransmitters, of course, are central in synaptic transmission, for stabilization of the forming synapse and to grant a bidirectional communication between neurons and other cell types (i.e., astrocytes, microglia, oligodendrocyte) through feedback systems in both adult and developing brains [48].

Finally, neurotrophins (NTs) are indeed the main regulator of synapse formation and function, as they strengthen robust and functional circuitries while preventing redundancies or pathological rewiring [49]. The NT family encompasses the nerve growth factor (NGF), the brain-derived neurotrophic factor (BDNF), NT-3, and NT-4/5 in mammalians (NT-6 and NT-7 in fishes) [50,51]. NT receptors are divided into two distinct classes. The p75 is the first receptor being identified. It shows a low affinity for all NT without a significant specificity and seems to be involved in the apoptotic pathway [52]. High-affinity receptors for NT are members of the tropomyosin receptor kinase (Trk) family. TrkA is the high-affinity receptor for NGF; NT-4/5 and BDNF are preferred ligands of TrkB, while NT3 binds to TrkC [51].

All NTs share a very high amino acid homology (approximately 50%) and are initially synthetized as pre-pro-proteins in the endoplasmic reticulum. The amino terminal contains, in fact, the signal for intracellular transportation and is cleaved to obtain the proNT, which undergoes post-translational modification in the Golgi apparatus. ProNTs can be processed to NTs both intracellularly and extracellularly, with the consistent contribution of both astrocytes and ECM [53] (Figure 1 and Figure 2). The intracellular pathway consists of proteolytic cleavage by the pro-protein convertase subtilisin kexin (PCSK), particularly the neuronal form PCSK1/2, which binds to specific recognition motifs of the pro-protein, leading to the mature NT that can be subsequently stored in vesicles and secreted [54,55]. Impaired extracellular proNTs processing and activation can compromise the stability of the synapse, as detailed below.

All NTs act as modulators of synaptic plasticity. TrkB activation has been shown to increase the density of presynaptic vesicles and the expression of vesicular VAMP1/2 and Syp, thus enhancing the exocytotic machinery. BDNF is practically ubiquitous in the CNS and is involved in neuronal survival, axon growth, cell morphology, induction of protein expression and adaptive plasticity, which is pivotal for both learning and memory formation [56]. Similarly, NGF has been shown to regulate mechanisms underlying development and energy homeostasis of NGF-dependent neurons [57], moreover to its well-established role in modulating synaptic plasticity of cholinergic neurons [58].

Levels of synaptic proteins have been investigated in models of neurological degenerative diseases by proteomic approaches to unravel their involvement in neurodegeneration. Dysfunctional levels of NTs, as well as SNAP-25, Syntaxin-1, Syp, PSD-95, MAGUK, and SHANK proteins, are involved in neurodegenerative diseases, such as Alzheimer’s disease (AD), Parkinson’s disease (PD), and dementia with Lewy bodies (DLB) [59]. Protein modification at the synaptic cleft are common pathways of neurodegeneration, preceding neuronal death and late-onset modifications in non-cell autonomous diseases. The complexity and similarity observed in the abovementioned neurodegenerative diseases corroborate the need for a systems biology approach [22,58]. The production of a holistic model that encompasses the complex, reductive, networks of proteins and metabolites belonging to distinct synaptic components (i.e., neurons, astrocytes, microglia, ECM and NVU), as well as to sub-cellular compartments (i.e., synaptic vesicles, mitochondria, etc.), can support a better understanding of cell–cell and cell–matrix-NVU interactions, as well as how these networks are affected under physiological or perturbed synaptic transmission conditions (adaptive and maladaptive plasticity) (Figure 1). For instance, such a model might allow predicting how the activation of a plasmatic protease, such as thrombin, could affect astrocytic and microglial activation, remodeling the ECM and ultimately influence the intracellular pathway of neuronal cells and proteins of the synaptic cleft (Figure 2).

## 3. Glial Cells

Synaptic plasticity is influenced by the three main non-neuronal resident cells: Astrocytes, microglia, and oligodendrocytes acting as a functional unit. The contribution of each cell-type will be described enlightening the peculiarities, possible networks within these elements and how they interact with the ECM and the NVU.

### 3.1. Astrocytes

Since the proposal of the tripartite synaptic model in 1999, astrocytes and their morphological variants and subtypes have been the most vastly investigated glial cells [5]. Indeed, astrocytes have a prominent role in synaptic plasticity: On one side, they, tightly enwrap neuronal cells and synapses (Figure 2) [60], and participate to the production and maintenance of the ECM; on the other hand, they are associated through their end-feet with the endothelium and pericytes in the NVU (Figure 1) [15]. In the penta-partite synaptic model, this cell-type could be considered as the cornerstone; the study of the related network with the other components could open a new field of experimental speculations.

The astrocytic influence on the synaptic cleft is required for CNS homeostasis [61]. For instance, the astrocytic Hevin interacts with the neurexin/neuroligin system to ensure synaptic clef stability (Figure 2) [62]. Astrocytes are hyperpolarized cells in the resting-state through the specific expression of inward rectifying potassium channels (Kir), particularly Kir 4.1 [63]. These channels finely expressed on the astrocytic membrane facing the synaptic cleft can reduce the potassium conductance during neuronal activity. The dysfunction of this mechanism, leading to improper neuronal membrane depolarization, has been found in various neurological diseases, such as epilepsy, multiple sclerosis (MS), amyotrophic lateral sclerosis (ALS), and Huntington’s disease (HD) [64,65]. Another channel expressed on the astrocytic membrane is the aquaporin-4 (AQ-4) that is involved in neuromyelitis optic spectrum disorders (NMOSD) [64]. AQ-4 water channel has been described on the astrocytic end-feet, and associated with Kir channels to control the osmotic regulatory role of these cellular domains; in fact, potassium uptake during neuronal activity generates an osmotic imbalance [66]. Furthermore, AQ-4 is the pivotal player in the proposed glymphatic mechanism of ECM debris removal through NVU and CSF active directional filtration (Figure 1) [17,18,67]. Considering these proteins not only as peculiar in astrocytic regulation but as paramount in the entire plasticity process, could account for novel strategies of targeted therapies to improve, for instance, the clearance of misfolded proteins (e.g., AD, PD). On the other hand, the AQ4-NVU interface could explain the immune-mediated phenomenon of NMOSD and implement novel strategies for adaptive synaptic regulation following the lost immune privilege.

Astrocytes are also important in neurotransmitters reuptake (GABA and glutamate) through specific transporters, thus modulating the concentration of these mediators and confining them to the synaptic cleft (Figure 2). The main glutamate transporters for astrocytes are glutamate transporter 1 (GLT1) and glutamate/aspartate transporter (GLAST) that have been shown to prevent glutamate over-excitation observed in several pathological conditions, such as trauma or epilepsy [68,69]. The confinement of glutamate in the synaptic cleft avoids glutamate spillover, and its reuptake is adjuvated by neuronal transporters, the excitatory amino acid carrier 1 (EAAC1) [1,70]. The activation of extra-synaptic metabotropic glutamate receptors (Figure 2) is responsible for the modulation of excitatory postsynaptic currents (EPSCs) [71] that can modify temporal and local integration of synaptic currents, thus affecting synaptic plasticity. Inhibitory modulation, on the other hand, is due to the GABA transporters (GAT), particularly GAT1 and GAT3, the first one shared with neurons, the latter being able to regulate the astrocytic intracellular concentration of calcium [72]. Moreover, astrocytic GAT3 can enhance the release of purinergic mediators (ATP/adenosine) in the hippocampus [72]. Modification of GAT and astrocytic calcium dynamics have been reported in a rodent model of behavioral disorders [73].

Another important feature of astrocytes at the synapse is the calcium-mediated release of molecules, once thought to be exclusively neuronal, called gliotransmission (Figure 2) [74]. Although there are controversial data regarding the astrocytic expression of proteins necessary for vesicular release, the dynamic regulation of synaptic transmission through astrocytic activity has been extensively proved [75,76]. Among the molecules acting as gliotransmitters, glutamate, GABA, D-serine, ATP and adenosine have been shown to control the basal tone and threshold of synaptic activity, surpassing the all-or-none law of the action potential-mediated neurotransmitters release [77,78]. Finally, astrocytes participate in the secretion of proNTs and their processing in the ECM [14]. Moreover, NTs synthesized in neurons and secreted as proNTs into the ECM can be rapidly internalized into perineuronal astrocytes via p75 mediated endocytosis. After internalization, they can undergo a recycling or activation process [1] (Figure 2).

One single astrocyte can enwrap about 120,000 synapses in rodents. A human astrocyte might unsheathe from 270,000 to 2 million synapses [79,80] that can be both excitatory or inhibitory, thus encompassing different neuronal circuits and eventually integrating them [80], enhancing both short-term (STP) and long-term (LTP) potentiation, or decreasing the glutamatergic tone with GABA or the purinergic release [81,82]. The cannabinoid (CB) system, furthermore, seems to be a signaling pathway by which activated astrocytes release glutamate and enhance the synaptic strength with both short-term [83] and long-term plasticity [84]. D-serine is also released by CB activation and stimulates the N-methyl-D-aspartate glutamate receptor (NMDAR) contributing to hippocampal LTP, the proposed main mechanism for memory formation and maintenance [85]. Besides, the calcium-binding protein S100b produced by astrocytes can induce neuronal firing in both trigeminal sensory nucleus and prefrontal cortex and might regulate cognitive flexibility and neuronal oscillations [86]. Intriguing is even the role of astrocytes in the regulation of sleep: Adenosine accumulation is associated with sleep homeostasis [87] and the astrocytic release of ATP/adenosine and glutamate can induce a transition between wakefulness and sleep [88,89]. A fascinating pathway that connects astrocytes to the vascular system through the ECM, particularly during sleep, is the abovementioned glymphatic system [67]. This hypothesis considers experimental data conveying a possible CNS lymphatic-like system mediated by astrocytes that is responsible for ECM clearance of accumulated molecules and control of metabolic supplies [90]. A failure of the glymphatic system has been related to toxin accumulation, such as amyloid-β (Aβ) [91] and misfolded proteins in the perivascular space of murine models of type-2 diabetes and AD, although a direct correlation between these findings and the cognitive deficits associated with human dementia are still debated [92].

Astrocytes are interconnected with gap junctions formed by connexins (Cx), Cx43 and Cx30 being the most expressed [93,94]. The intercellular diffusion of ions, transmitters, and small molecules connects networks formed by hundreds of astrocytes, not with a mere neighboring principle but with functional purposes [95]. The cortical spreading depression (CSD), one of the main explanatory mechanisms of typical migraine aura, could depend on Cx-based astrocytic syncytia [96]. CSD, in fact, could involve waves of synchronous astrocytic activation (through their gap junctions), with a slow-propagating neuronal firing followed by reactive hyperpolarization in limited cortical area, thereby causing the transient neurological positive and negative focal symptoms characterizing the migraine aura [96]. As a clinical proof of concept, a gap junction inhibitor, tonabersat, was effective in both CSD inhibition and migraine aura frequency reduction [97]. Altered expression of Cx at the gap junction can also influence glucose and lactate supply and synaptic transmission [88,98]. Simple as it may sounds, there is still debate on the glucose/lactate coupling between astrocytes and neurons [99,100] (Figure 2).

Astrocytes are highly sensitive to plasticity processes and may act with structural changes involving their cytoskeleton and different expression of the glial fibrillary acidic protein (GFAP). These cells have a high motility rate of morpho-functional re-shaping in a timescale of minutes [101]. Sustained NMDAR activation can increase glutamate release from astrocytes even 1 h after neuronal stimuli, widening the time window for synaptic plasticity [102]. Astrocytes are practically able to store synaptic information over time and can, in turn, modulate late-onset synaptic plasticity of the same synaptic pathway or related neuronal circuits, reinforcing the interdependency of neuron-astrocyte processes. The features of astrocytes vary across brain areas and are dynamically connected to neuronal specialization, with their diversity being even more complex in different species [8,79].

All the discussed regulatory functions of astrocytes in synaptic plasticity make puzzling and intriguing the fact that these cells do not cover all synapses [60,103]. The extent of synapse sheathing varies from 15% for mossy fibers of granule cells to 85% for climbing fibers in the cerebellum and 90% for excitatory synapses in the somatosensory cortex of adult mice [104,105]. Differences between these synapses (as well as the relative involvement of other glial cells, nonresident-cells and matrix components in the maintenance of plasticity) corroborate the need for a multi-cellular interconnected model that could allow the understanding of a disease by analyzing the system as a functional unit. Moreover, it has been shown that synapse enwrapping can be dynamically induced by neuronal activity and by physiological conditions like nutritional state (satiety or starvation) [103,106,107].

### 3.2. Microglia

Microglia can rapidly sense differences and homeostatic perturbations by scanning other cells (astrocytes and neurons) and the brain ECM (Figure 1) [108], and surveying the environment for pathogens and autologous debris (phagocytic function) (Figure 2) as the first cellular line in the innate immune response [14,109,110,111]. In the last scenario, microglia can mediate the loosening of BBB and the secondary immune reaction [112]. All these functions are possible through a highly variable profile of gene expression and cell morphology [111]. In particular, microglial cells show the capability to actively assist not just the elimination (as thought considering their scavenger role), but practically the formation and/or relocation and reinforcement of synapses (e.g., maturation of excitatory synapses), by sensing the environmental pabulum for signaling molecules and re-wiring the circuitry following these biochemical instructions. Their role is ubiquitous in the CNS [10].

Their scavenger role has practically led to the strong parallelism between microglia and the macrophage system in other tissues. However, microglia is an ontogenically distinct population of the phagocyte system, with a different embryonic origin, compared to resident macrophages of other tissues [113]. For instance, there is not a continuous supply of microglia precursors from the general circulation (monocytes) and these cells renew themselves, slowly, in the mammalian brain maintaining a certain epigenetic memory of the environmental modifications [114,115]. Moreover, microglia bodies are relatively immobile, stretching highly dynamic elongations to scan the entire CNS in the timescale of hours [116]. This evidence and the aforementioned connection between microglia and the system gives the opportunity to enlighten the peculiar mechanisms that could be further studied to understand the main modifiers of synaptic plasticity. The classical macrophage-related categorization of M1 (pro-inflammatory phenotype) and M2 (resting or anti-inflammatory) phenotypes, is in contrast with transcriptomics studies showing multiple microglia responses with multifaceted polarization states [117,118]. The main activity of these cells does not seem to be the scavenging, as initially supposed. Their relevance to synaptic plasticity is linked to their capacity to sense the functional state of synapses, ECM and vascular compartment, and communicate with other resident cells [119]. This specialized function is based on high-density surface receptors (called sensome) to detect both physiological (cognitive stimulation, diet, physical exercise) or pathological stressors [108,120]. The main microglia receptors allow the communication with neurons, through the purinergic receptors family (e.g., P2XRs, P2YRs) [82] or the widely studied neuronal chemokine (fractalkine) receptors (CX3CR1) [121]. They also mediate the immune system and the NVU through the complement receptor CR3 [14], and activate the phagocytosis response by triggering the receptor expressed on myeloid cells 2 (TREM2) and DNAX-activation protein of 12 KDa (DAP12) [122,123].

These sensors have been shown to contribute to synaptic plasticity, neurogenesis, myelination, and blood vessel formation [124]. Concerning the synaptic plasticity, microglial processes establish contacts with neurites and synapses, particularly through the neuronal ligand fractalkine or the complement system C1q and C3, even if the exact mechanisms have not been completely elucidated [119,125]. It has been suggested that microglia could enwrap small portions of axons (a mechanism called trogocytosis) to limit and guide their growth and to eliminate presynaptic structures [126] (Figure 2). It has also been debated whether microglia can phagocytose dendritic spines or can partially surround them [126,127]. The interposition of microglial cells in the synaptic cleft [128], and the ability of their filipodia to sustain dendritic spine formation and relocation on the dendritic shaft, are fascinating features of microglia-guided synaptic plasticity (Figure 1) [126]. Furthermore, it has been shown a close contact between the axon proximal segment and microglia, proposing an unknown mechanism for the formation, interruption, or elimination of synaptic connections [129].

Another paramount role during CNS development is the microglia-dependent synaptic elimination, essential for the correct wiring of the system, particularly studied in the visual cortex [119]. The interaction with the synaptic cleft has been supported by the evidence of PSD-95 in microglial specialized processes both in vitro and in the mouse cerebral and hippocampal cortex [122]. While the observation of trogocytosis has been demonstrated for the axonal portions, the engulfment of dendritic spines has been always shown as partial, by the endurance of a connection with the dendritic shaft through the neck of the spine [126]. Importantly, CXCR1 and CR3, pivotal in a previously proposed neuro-immune network [14] in the presynaptic region seem to be responsible, respectively, for synaptic and axonal enwrapping with an activity-dependent elimination in certain brain areas [130,131]. The postsynaptic modifications of dendritic spines could be indirect, with the enwrapping or interposition of microglial processes and functional deprivation, as speculated in a mechanism of interfering plasticity shown during microglia activation by lipopolysaccharide (LPS) [128]. Microglia can modulate synaptic plasticity by production and secretion of molecules, such as ECM components, NTs (i.e., BDNF), endo(e)CB, cytokines (e.g., tumor necrosis factor α -TNFα-), micro-ribonucleic acids (microRNAs) by surface-anchored release or the formation of extracellular vesicles (EV) [125]. Microglial BDNF acting through TrkB neuronal receptor seems to be involved in the switch of GABA from an excitatory to an inhibitory molecule in adult neurons [132]. The release of microglial BDNF is induced by P2 × 4 activation in a model of neuropathic pain; while it was shown that EVs containing eCB are released by rat microglia exposed to ATP [132,133]. Nonetheless, the secreted BDNF seems to be important for the presynaptic vesicular glutamate transporter 1 (vGLUT1) expression [134]. Furthermore, platelets are also able to release BDNF during pathological processes (such as stroke) that allows their activation in the NVU as well as glial activation [112]. These data associate the microglia with the previously described NTs survival network and the limitation of cell-based approaches considering neurons and astrocytes as the main regulators of NTs in CNS pathophysiology. Expression of NMDAR and AMPAR and their ratio could be modulated by the astrocyte/microglia crosstalk and by microglial TNFα release in the maladaptive plasticity [135]. Eventually, the activation of stress responses mediated by microglial CR3 seems to induce LTD [136].

Overall, these data show an interaction between the microglial system and multiple levels of synaptic plasticity. The mentioned pathways are strongly associated with the penta-partite model and pivotal in the development of systems biology approach to the adaptive/maladaptive tuning. The activation of both astrocytes and microglia in maladaptive plasticity and various neurological diseases, known as reactive gliosis [1,53,137], will not be discussed in this review.

### 3.3. Oligodendrocytes

Oligodendrocytes are the myelin-forming elements of the CNS. They develop from the oligodendrocytes precursors cells (OPC). OPCs are important in CNS development but, unlike other progenitors, remain abundant in the adulthood and maintain the ability to modify the state of the white matter both in physiological (learning, normal aging, experience-based system rewiring) and pathological conditions (e.g., MS and NMOSD) [138]. OPCs express the proteoglycan marker neural/glial antigen 2 (NG2) cells. The proliferation and differentiation of these cells are mediated by various growth factors and hormones. Platelet-derived growth factor (PDGF) seems to be the most powerful inducer of OPC proliferation mediated by neighboring cells (neurons and astrocytes). PDGF, however, acts in association with ECM molecules and their cellular integrins (the phosphorylated form of α_V_β_3_ associated with PDGF receptor) to exert its mitogenic activity, with the relevant contribution of Tenascin-C and NG2 (Figure 1) [139]. These relations make OPCs relevant in the plasticity of the CNS with the ECM homeostasis.

The oligodendrocyte lineage proliferation, differentiation and survival, however, can be influenced also by neuregulins, NT-3 and NGF, further validating their role in synaptic plasticity [140,141]. Apart from their role in myelin and ECM homeostasis, novel interest is shown in the electrical signaling between oligodendrocytes and neurons. OPCs express a variety of neurotransmitter receptors, such as AMPAR, NMDAR, GABA, and acetylcholine (Ach) receptors [142] and voltage-gated channels (i.e., sodium, potassium, and calcium channels) that could in principle modulate the surrounding neuronal activity [138]. Moreover, OPCs are the only known glial cells to form synapses directly with glutamatergic neurons in both gray and white matter regions and with GABAergic neurons in the gray matter [143,144,145]. Unlike neurons, however, OPCs seem to participate only as postsynaptic terminals and their glutamatergic connections develop along with normal synapses in the surrounding neurons, while GABAergic signaling seems to gradually switch from synaptic to extra-synaptic [144]. The synaptic connection between OPCs and neurons appears to be counterintuitive, given the high mobility of OPC elements that continually reshape (forming and dissolving) synapses in their migratory pathway. The formation of these energetically expensive transient synapses allows the OPCs to monitor axonal activity in the neighboring neurons, guiding oligodendrocyte maturation and myelin synthesis. In fact, tetrodotoxin (TTX), a sodium channel blocker, can inhibit myelination [146], while stimulation of neural circuits even in adulthood can stimulate OPC proliferation and differentiation along the active pathways [147,148]. These synapses, however, can provide neurotransmitters, neurotrophic factors and integrins-mediated intercellular signaling, thus contributing to modulation of axonal outgrowth and/or neuronal excitability.

Oligodendrocytes, although maintaining the ability to regenerate, are long-lived cells, their lifespan apparently is independent from the brain area or the grade of axon myelinization [149] (Figure 1). New oligodendrocytes could replace myelin loss for physiological turnover and tend to accumulate with the time [150]. Myelination occurring during adulthood, however, seems to have different morphology, with a high density of shorter internodes [149]. The consequence is that the average internode length diminishes and the number of nodes per volume increases with aging, but the functional consequences of this remodeling are unknown and difficult to predict [151]. Behavioral phenomena should be expected in networks with rapid and synchronous information processing, such as auditory and visual systems [152]. In the auditory system, evidence suggests that synchronization on microseconds timescale is achieved through dynamic regulation of internode length and thickness [153]. Myelin synthesis as a proper synaptic plasticity mechanism can be triggered by a specific learning task, as demonstrated by myelin formation during motor-skill learning experiments [154] and the opposite was shown by the inhibition of a new motor-task acquisition by blocking new myelinization [147]. When myelination was blocked by conditional deletion of myelin regulatory factor, blocking the OPC differentiation into mature oligodendrocytes, animals were unable to learn new motor skills, but they were able to recall previous ones [147]. The role played by oligodendrocytes in synaptic plasticity has been neglected for decades, and substantially limited to demyelination processes [155]. A systems biology model of adaptive/maladaptive plasticity including this cellular component should help to obtain a more comprehensive framework of their involvement in acute (e.g., stroke) or chronic (e.g., corticobasal degeneration) CNS diseases.

## 4. ECM and NVU

Synaptic plasticity processes require structural and functional modifications, including shape, density, formation, and elimination of synapses [2]. The functional modifications have been mostly related to the neuron-glia interactions, like neurotransmitters secretion, receptors expression, activity-dependent synaptic plasticity (e.g., LTP, LTD, myelination). This physiological plasticity allows the circuitry to retain and reinforce the connections and store information [156,157]. The ECM and NVU, on the other hand, participate actively in the structural changes and mediate the bioavailability of nutrients, cytokines, molecular mediators (e.g., NT, transmitters, integrins ligands) that are fundamental for the correct sequence of functional modifications [14,112,157]. The major structural builder of these functional scaffolds could not be identified, as all CNS resident cells are involved in producing or re-shaping them both in health and diseases (Figure 1).

### 4.1. ECM

The molecular composition of ECM in the CNS has been deeply investigated to elucidate the intricate structure of this functional scaffold [11,12]. The major components of neural ECM are proteoglycans (e.g., brevican, neurocan), interacting with collagen, glycoproteins (e.g., tenascins) and hyaluronic acid (HA) synthesized in a different ratio by both glia and neurons (Figure 1). Unbiased mechanisms activated progressively during development and in physiological conditions in the adult brain, reshape the synaptic cleft through these ECM molecules.

Morpho-structural modifications of the ECM during CNS injury, aging and reactive astrocytosis have been studied for their implication in neuroinflammation, neurodegeneration and the maladaptive synaptic plasticity.

Elegant experiments were made to discriminate the astrocytic and neuronal ECM contribution using co-cultures of neurons and astrocytes obtained from quadruple knockout mice for Tenascin-C/R, brevican and neurocan. These co-cultures showed reduced production and organization of the ECM, with lowered neuronal activity. The integration of wild-type astrocytes did not rescue the neuronal phenotype [158] and co-cultures containing either quadruple knockout astrocytes or neurons showed a reduced number of synapses (after an initial transient increase of synaptic formation in co-cultures containing mutant astrocytes) [158]. These in-vitro data were partially complying with the in-vivo studies conducted with the same animal models, showing mild defects of ECM deposition and the replacement of the lost components with fibulin 1/2 during development [159]. Understanding the relevance of cell-type-specific ECM components could be fascinating, like the astrocytic hevin (or its antagonist SPARC) and thrombospondins, although all cellular elements seem to act all together as a functional unit in synaptic plasticity [3,160].

Furthermore, MMPs and ADAMTS (A Disintegrin And Metalloproteinase with thrombospondin motifs) proteases are secreted by both glia and neurons and can reshape the ECM in response to external or internal perturbations of the homeostasis [12]. These speculations have made the study of the ECM promising for novel therapeutic approaches, although still practically controversial, possibly for the delicate balance of this functional scaffold [161,162].

The PNNs, a specialized form of ECM, seem to be key elements in synaptic stability, creating a functionally permeable barrier that allows or limits the formation of new connections between neurons [156]. Furthermore, PNNs retain synaptic signaling molecules (e.g., semaphoring-plexin system) that in the adult brain prevent the formation of unfit neuronal circuitries [163]. ECM has a different composition in the developing brain with a high level of expression of matrix components reported before the postnatal synaptogenesis peak, suggesting the key role played by the interstitial matrix for the formation of immature synapses [164]. Aggrecan, also known as chondroitin sulfate proteoglycan (CSPG)-1, seems to be particularly expressed on the surface of neurons in correspondence to the loci of forming synapses [165].

Considering their role in synaptic stability, PNNs were shown as fundamental elements for memory maintenance [166]. CSPGs, major components of the PNNs, were increased in the amygdala of fear-conditioned animals and were associated with the protection of long-term memory traces, resistant to extinction. The formation of these memories seems to coincide with the organization of CSPGs into PNNs, related to the closure of a fragile period of the synaptic developmental plasticity, defined “critical period” [166].

The increased expression of CSPGs, Tenascin-C, Tenascin-R, and HA can regulate axon elongation mediated by reactive astrocytes [167,168]. CSPGs are selectively overexpressed in maladaptive plasticity. Astrocytes produce neurocan and phosphacan, following cerebral cortex injury, while brevican or versican expression is not increased [169]. Moreover, the induced modification of ECM through heparinase or chondroitinase ABC (ChABC) overcomes the limitation of neurite outgrowth following glial scar formation [169,170]. The specific expression of CPSGs seems to depend on the specific neurological disease. An overexpression of brevican in the frontal cortex was reported in AD [171]. Oligomers or fibrillary Aβ peptides (not the monomers) were able to bind brevican core protein [171]. Animal models of AD, when treated with ChABC, showed a certain degree of LTP function in the hippocampus and short-term memory formation with a higher synaptic density. The accumulation of brevican seems to be due to astrocytic deposition and leads to synaptic maladaptive plasticity [171].

The maturation of NTs (i.e., NGF and BDNF) can occur as abovementioned in the ECM. In this case, proNTs are processed by serine proteases like plasmin and MMPs, particularly MMP-7 and MMP-9 [55,172]. Plasmin, normally produced in its inactive form, the zymogen plasminogen, can be activated by the protease tissue plasminogen activator (tPA). Plasminogen seems to be exclusively expressed by neurons [173].

Cell adhesion molecules (CAM) are pivotal for the interaction between cellular elements and ECM. In particular synaptic CAM (SynCAM), neuroligins and hevin (involving both neurons and astrocytes) have demonstrated a role in the developmental synaptic plasticity, and more recently, also a member of the leucine-rich repeat transmembrane (LRRTM) proteins [174,175]. LRRTM members are transmembrane proteins, interacting with the ECM. They bind neurexins and induce presynaptic differentiation playing a role in the regulation of receptor composition [175,176]. The deficiency of LRRTM leads to the loss of different types of synapses with parallel impairment of pre- and post-synaptic components of the cleft [177,178]. LRRTM is able to bind neuroligin, but its role could influence different types of synaptic sprouting, through the different components of the surrounding ECM. However, the role of this protein family in ECM-mediated synaptic plasticity needs further investigation. Furthermore, the neural cell CAM (NCAM) is an important player in visual cortex development [179]. In particular, the visual stimuli could induce a polysialylation of NCAM (which account for 95% of CNS protein polysialylation) and enhance the homophilic interactions across the synapse [179]. Similar modifications are found in SynCAM, suggesting another possible mechanism to organize specialized synaptic composition [180]. The physiological function of polysialylation is to enhance hydration and volume size of the molecule, thereby increasing the distance between the cell membranes of polysialylated NCAM-carriers and following regulation of cell–cell interactions [181]. Specific patterns of polysialylation are characteristic of developmental and adult brains, with almost overlapping NCAM expression and a small fraction of SynCAM polysialylated after birth [181]. Moreover, polysialylation of SynCAM seems to be confined to few brain areas and could be found also on OPCs [181]. These data are in accordance with the role of OPCs in synapse maturation and ECM homeostasis.

### 4.2. NVU

Finally, we need to consider the complexity of the NVU. This component allows the CNS homeostasis, metabolic supply, and immunological privilege, being regulated by a fine tuning mode between the BBB elements (endothelial cells, astrocytic end-feet, pericytes, and the basal membrane), neurons, glia and interstitial ECM [14,182].

The immune privilege of the CNS applies to both brain and spinal cord (through the BBB homologous, blood-spinal cord barrier) [183] and can be maintained via the innate immune properties of resident microglia, constantly scanning the environment to detect endogenous perturbations or external pathogens and quickly restoring local homeostasis, thereby avoiding or confining the damage [184]. Neuroimmune regulators (NIRegs) are a group of signaling proteins that are expressed on both glia and neurons, and act to limit the immune activation [185]. Microglia remain in a resting state by interacting with NIRegs (e.g., CXCL1, CD200, and CD47) on other cells. NIRegs nonetheless inhibit complement activation through CD59, CD46 and factor H (FH) [185]. Furthermore, cytokine signaling can be physiologically modulated, by the constitutive levels of the suppressor of cytokines signaling (SOCS) that inhibits the Janus kinase (JAK)/signal transducer and activator of transcription (STAT) intracellular pathway [185].

Thrombin is one of the main activators of neuro-immune responses and is strictly linked to the NVU. This protease can exert both adaptive and maladaptive plasticity modifications on neurons and glia [186,187] depending on its concentration, by interacting with the proteinase-activated receptors (PARs). PARs are G-coupled receptors, with four recognized members [9,188] demonstrated on neurons, glia, endothelial, and immune cells. PAR-1 can be canonically activated by thrombin, activated coagulation factor X (FXa), MMP-1 and plasmin (also important as ECM reshaping proteins). The activation is mediated by proteolytic cleavage and the tethered ligand exposure [189].

PAR-1 canonical activation seems to be neurotoxic and is achieved with pathologically high concentrations of proteases [190] activating a guanosine triphosphatase (GTPase), the rat sarcoma protein (Ras), the related protein A (RhoA). PAR-1 biased agonism, instead, can be achieved with a controlled thrombin response, complexed with the activated protein C (aPC), which binds the endothelial PC receptor (EPCR). This induces a different proteolytic activation on PAR-1 stimulating another GTPase, the Ras-related C3 botulinum toxin substrate 1 (Rac-1), which interacts with βarrestin-2 and disheveled-2 [191]. Other PARs seem to show secondary functions, particularly PAR-3 which lacks intracellular domains [192], thus being unable to activate directly a G protein. Indeed, it could act as a cofactor and form an heteromeric complex with other members of the family to modulate intracellular transduction [193]. The complement factor C4a seems to act as an untethered ligand of both PAR-1 and PAR-4, leading to intracellular activation of phospholipase C (PLC) and intracellular calcium release [194], supporting the relevance of neuro-immune modulation of the NVU.

Serpins and thrombomodulin are also considered NIRegs and could reduce the toxicity of thrombin on the CNS by preventing the canonical activation of PAR-1 [195]. LPS administration can stimulate production in the hippocampus of microglial inflammatory factors and the expression of coagulation factors, probably depending on thrombin signaling activation [196]. The relevance of these factors has been proved in neurological and psychiatric diseases [14].

The role of pericytes, on the other hand, was largely ignored, although it was reported that a mutant mouse model with pericyte deficiency showed an significant increase of BBB permeability to both water and solutes of low and high molecular mass [197]. This increased permeability was due to endothelial trans-cytosis. Moreover, it has been shown that pericytes could alter gene expression patterns of the endothelium and induce the polarization of astrocytic processes into the end-feet surrounding the vessel [197]. Vascular changes involving pericytes and preceding the striatal and cortical changes were described also in a mouse model of HD, and changes of these cells were found in post-mortem HD human brains [198].

The CSF dynamic exchange of solutes with a directional flow from the arterial perivascular space into the brain parenchyma and the consequent venous drainage was recently added to the regulating functions of the NVU. In vivo studies demonstrated an active flow from the cisterna magna to the subendothelial space, regulated by astrocytic end-feet of the so-called glial-limiting membrane [17,92]. This process implicated in the CNS waste clearing (Figure 1), seems to be particularly active during sleep, with a cyclic increase of the interstitial space and lower noradrenergic tone mediated by the *locus coeruleus*. The increased ECM space alters the synaptic transmission and contributes with the arterial pump to the CSF influx and interstitial solute exchange during wakefulness-sleep rhythms [91] and possibly during sleep phases transitions. Although there are increasing data concerning the physiology of the glymphatic system and the involvement of the NVU in supporting its role in consciousness and CNS pathology (traumatic, vascular, autoimmune, or degenerative), a translational approach is still lacking [18,67,90,92,199,200].

## 5. Conclusions and Perspectives

Recent data suggest that a paradigm-shift in CNS studies is mandatory. To understand CNS complexity, novel experiments should focus on the functional cooperation between several cellular, sub-cellular and molecular components in synaptic plasticity. This can be achieved by a systems biology approach. Neurons, although paramount for synapse functioning, are not able to develop, reshape and reinforce the circuitry of the brain on their own.

The support of the glia is essential for trophic factors and neurotransmitters modulation, axon myelinization, and synapse re-localization and elimination. On the other hand, the structural scaffold of the ECM consistently regulated by all resident cells acting as a functional unit can be pivotal in both developmental and several physiological changes. Moreover, the ECM is modified by pathophysiological processes of CNS diseases due to its competence in NTs storage, axon guidance, circuitry protection and intercellular communication. This synaptic model also accounts for the role exerted by the NVU in the metabolic supply, BBB maintenance, glymphatic system, coagulation and immune-system intervention in both adaptive and maladaptive plasticity. NVU and its peculiar structure support selective and controlled exchange between CNS and the blood flow, with precise rules and the possibility to modulate synaptic transmission.

Here we have enlightened the state-of-the-art evidence of the main pathways that should be considered if we want to develop a comprehensive view of synaptic function under physiological and pathological modifications. The increasing amount of omics data (i.e., genomic, epigenomic, transcriptomic, proteomic, metabolomic, connectomic) gained us with an extremely complex picture of the molecular events underlying perturbed conditions linked to neurological and neurodegenerative disorders. On the other hand, we need to organize these big-data into a dynamic, integrative model that takes into account not only the molecular networks but also their relative distribution between cellular and sub-cellular elements. Indeed, modular systems biology is a strategy that allows structuring the information about complex biological processes to obtain modular and mathematical/computational models that may favor the identification of the key steps of the process, as well as the prediction of how the molecular events of the network will respond to specific perturbations of the system. The perspective is to be able to comprehend the regulatory logic of the complex molecular network, which belongs to different cellular and non-cellular domains (neurons, astrocytes, ECM, and NVU). A clear understanding of these mechanisms, through an iterative process of computational and experimental validation, could lead to the design of new drugs and innovative effective treatments for neurological diseases.
